# Investigation of a *Listeria monocytogenes* Chromosomal Immigration Control Region Reveals Diverse Restriction Modification Systems with Complete Sequence Type Conservation

**DOI:** 10.3390/microorganisms11030699

**Published:** 2023-03-08

**Authors:** Phillip Brown, Sangmi Lee, Driss Elhanafi, Wilhelm Tham, Marie-Louise Danielsson-Tham, Gloria Lopez-Valladares, Yi Chen, Mirena Ivanova, Pimlapas Leekitcharoenphon, Sophia Kathariou

**Affiliations:** 1 Department of Plant and Microbial Biology, North Carolina State University, Raleigh, NC 27695, USA; 2 Department of Food and Nutrition, Chungbuk National University, Chengju 28644, Chungbuk, Republic of Korea; 3 Biomanufacturing Training and Education Center, North Carolina State University, Raleigh, NC 27695, USA; 4 School of Hospitality, Culinary Arts and Meal Science, Örebro University, 702 81 Örebro, Sweden; 5 Division of Microbiology, Center for Food Safety and Applied Nutrition, Food and Drug Administration, College Park, MD 20740, USA; 6 Research Group for Genomic Epidemiology, National Food Institute, Technical University of Denmark, 2800 Lyngby, Denmark; 7 Department of Food, Bioprocessing and Nutrition Sciences, North Carolina State University, Raleigh, NC 27695, USA

**Keywords:** *Listeria*, restriction modification system, immigration control region, whole genome sequencing, chromosomal hotspot

## Abstract

*Listeria monocytogenes* is a Gram-positive pathogen responsible for the severe foodborne disease listeriosis. A chromosomal hotspot between *lmo0301* and *lmo0305* has been noted to harbor diverse restriction modification (RM) systems. Here, we analyzed 872 *L. monocytogenes* genomes to better understand the prevalence and types of RM systems in this region, designated the immigration control region (ICR). Type I, II, III and IV RM systems were found in 86.1% of strains inside the ICR and in 22.5% of strains flanking the ICR. ICR content was completely conserved within the same multilocus sequence typing-based sequence type (ST), but the same RM system could be identified in diverse STs. The intra-ST conservation of ICR content suggests that this region may drive the emergence of new STs and promote clone stability. Sau3AI-like, LmoJ2 and LmoJ3 type II RM systems as well as type I EcoKI-like, and type IV AspBHI-like and *mcrB*-like systems accounted for all RM systems in the ICR. A Sau3AI-like type II RM system with specificity for GATC was harbored in the ICR of many STs, including all strains of the ancient, ubiquitous ST1. The extreme paucity of GATC recognition sites in lytic phages may reflect ancient adaptation of these phages to preempt resistance associated with the widely distributed Sau3AI-like systems. These findings indicate that the ICR has a high propensity for RM systems which are intraclonaly conserved and may impact bacteriophage susceptibility as well as ST emergence and stability.

## 1. Introduction

*Listeria monocytogenes* is a Gram-positive facultative intracellular pathogen and the causative agent of the severe foodborne disease listeriosis with severe health complications including septicemia, meningitis and stillbirths and case fatality rates of approx. 15% [1]. At especially high risk are pregnant women and their fetuses, the elderly and those who are immunocompromised [2]. *L. monocytogenes* typically causes human illness through the contamination of processed, ready-to-eat foods, making the food processing environment (FPE) a vital reservoir of concern for the control of this pathogen [2,3]. Industrial sanitizers such as quaternary ammonium compounds are extensively used in the FPE, but *L. monocytogenes* FPE isolates are frequently resistant to benzalkonium chloride and other quaternary ammonium compounds [4,5,6].

To further control *L. monocytogenes* in FPEs, alternative antimicrobial agents have been developed and subsequently approved for use by the United States Food and Drug Administration (FDA), including the use of commercial bacteriophage cocktails such as Listex^TM^ and ListShield^TM^ [7,8]. However, similarly to sanitizers, bacteriophage resistance can be encountered among FPE isolates [9,10,11,12,13,14]. Bacteriophage resistance in *L. monocytogenes* commonly involves the loss of wall teichoic acid (WTA) decorations such as *N*-acetylglucosamine, rhamnose or galactose [14,15,16].

*L. monocytogenes* is partitioned into four lineages, with lineage I (serotypes 1/2b, 3b, and most strains of serotype 4b) and lineage II (serotypes 1/2a, 3a, 1/2c, 3c) accounting for the majority of human listeriosis, while lineage III and IV strains remain uncommon in human illness and are highly diverse [17,18,19]. While 14 *L. monocytogenes* serotypes have been identified, most human cases of listeriosis typically involve serotypes 1/2a, 1/2b and 4b [20,21]. Serotype 4b accounts for approx. 50% of all human listeriosis cases and includes all of the major *L. monocytogenes* hypervirulent clones, i.e., clonal complex (CC) 1, CC2, CC4, and CC6 [22]. Serotype 4b strains are noticeably less likely to exhibit phage resistance than strains of other serotypes, and loss of WTA decoration with galactose has adverse impacts on virulence [23,24]. Furthermore, putatively functional CRISPR systems have been identified in all major *L. monocytogenes* serotypes except for serotype 4b [25,26,27].

With their propensity to maintain intact WTA glycosylation profiles and their lack of CRISPR systems, serotype 4b strains may rely on restriction modification (RM) systems as their most important strategy to combat phage infection. RM systems are categorized into four major types (I, II, III and IV) and protect against foreign DNA (e.g., bacteriophage, plasmids and transposons) by cleaving invading DNA at target sequences that lack appropriate methylation [28]. Type I, II and III systems mediate both restriction and methylation of specific target DNA sequences, while Type IV systems lack a methylase and instead cleave modified, typically methylated DNA [29]. This targeted cleaving of DNA by RM systems can confer phage resistance by blocking the normal lytic cycle before the phage is able to replicate inside the cell.

Type II RM systems were previously identified in serotype 4b strains, including in the hypervirulent clones CC1 and CC6 [30,31,32], with the CC6-specific RM system LmoH7 conferring temperature-regulated resistance to all tested wide host range phages [31]. With the exception of LmoH7, these RM systems were localized between *lmo0301* and *lmo0305* homologs [32], identified as one of the major chromosomal hotspots in *L. monocytogenes* [33]. Type II RM systems were also found to be the most common RM system type in an analysis of 318 genomes of *L. monocytogenes*, with GATC being the most common recognition motif [34]. Previously, a region of the *Escherichia coli* chromosome, which included the type I RM system EcoKI and type IV methylation-dependent Mrr system, as well as a chromosomal region of the plant pathogen *Xylella fastidiosa* which harbored type I RM systems, were termed the “immigration control region” (ICR) [35,36]. Due to the high prevalence of RM systems in this chromosomal hotspot (*lmo0301*-*lmo0305*) in *L. monocytogenes*, we deemed it appropriate to also refer to this region as ICR [37]. In this study, our objective was to harness whole-genome sequence data from a large panel of diverse strains of *L. monocytogenes* to investigate the genomic content and distribution of RM systems in the ICR and to assess the possible implications for susceptibility to lytic phages.

## 2. Materials and Methods

### 2.1. Whole-Genome Sequence Analysis and Nucleotide Alignments

A total of 872 *Listeria monocytogenes* strains were investigated in this study (Table S1). Accession numbers for publicly available sequenced genomes are provided in Table S1. Strain serotype designations, sequence types (STs) and CCs were determined in silico by the BIGSdb PubMLST database hosted by the Institut Pasteur (https://bigsdb.pasteur.fr/listeria/ accessed on 4 March 2023) [38]. Investigation of immigration control region (ICR) content was completed with the Bacterial and Viral Bioinformatics Resource Center (BV-BRC) [39] using the Genome Browser function. Annotation of genes found in this region, including RM system nomenclature, was conducted using RAST [40], BLASTp hosted by the National Center for Biotechnology Information (NCBI) Basic Local Alignment Search Tool (BLAST) (https://blast.ncbi.nlm.nih.gov/Blast.cgi accessed on 4 March 2023) [41], HHpred hosted by the Max Planck Institute for Biology (https://toolkit.tuebingen.mpg.de/tools/hhpred accessed on 4 March 2023) [42] and REBase (http://rebase.neb.com/rebase/rebcit.html accessed on 4 March 2023) [43]. Nucleotide alignments were completed using ClustalW version 2.0 (http://www.clustal.org accessed on 4 March 2023) [44]. Statistical analyses, including Chi-squared distributions, linear regression (R-Squared) and unpaired *t*-tests, were completed using JMP Pro 17 (SAS Institute, Cary, NC, USA).

### 2.2. Generation of Minimum Spanning Trees and Gene Annotations

Minimum spanning trees were generated using BioNumerics version 8.1 (https://www.bionumerics.com accessed on 4 March 2023) (Applied Maths NV). The seven-locus multilocus sequence typing (MLST) scheme was used to group strains into sequence types (STs).

## 3. Results and Discussion

### 3.1. The Immigration Control Region Frequently Harbors Restriction Modification Systems That Exhibit Serotype-Dependent Trends

**Figure 1:**
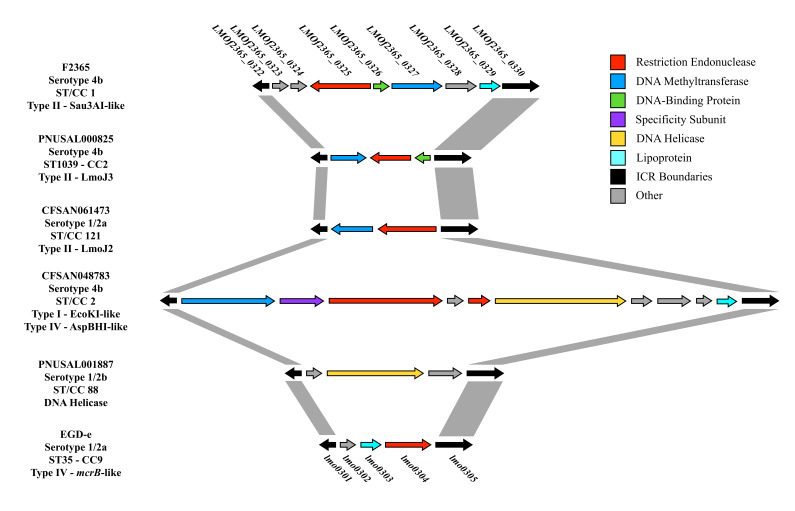
Genetic organization of the immigration control region (ICR) in six *Listeria monocytogenes* strains. Conserved boundaries of the ICR are indicated with black arrows and with gray connections. Arrows are color-coded based on gene annotations, which are provided in the key. Genomes were selected in order to visualize the most abundant and diverse content found in the ICR. Gene names above F2365 (e.g., *LMOf2365_0322*) and EGD-e (e.g., *lmo0301*) denote RefSeq locus tags for these reference *L. monocytogenes* strains.

The majority of chromosomal diversity in *L. monocytogenes* appears to be located in nine hypervariable hotspots, and restriction modification (RM) systems have been frequently detected in three of these nine chromosomal locations: *lmo0293*-*lmo0296*, *lmo0301*-*lmo0314* and *lmo1096*-*lmo1126* [33]. Previously, all tested strains of the ubiquitous, ancient and hypervirulent clone CC1 (formerly designated Epidemic Clone I, ECI) were found to harbor a Sau3AI-like RM system targeting GATC sites in the region between *lmo0301* and *lmo0305* [30,45], with this region hereafter deferred to as the immigration control region (ICR), as indicated above. As is typical of type II RM systems, this Sau3AI-like RM system included a gene for a restriction endonuclease and its cognate cytosine methyltransferase (Figure 1). Subsequent studies revealed that certain strains of other serotypes and genotypes also harbored the same Sau3AI-like RM system at the same location [30,34]. Furthermore, two unrelated type II RM systems, LmoJ2 and LmoJ3, specific for GCWGC (W = A or T) and GCNGC (N = A, T, G or C), respectively, were identified in the ICR in other strains [32]. The diversity in the ICR can be readily visualized via the comparative genomic analysis of the region harboring the Sau3AI-like cassette in the ST1 (CC1) strain F2365 and other genomes (Figure 1).

To further characterize the *L. monocytogenes* ICR, we screened in silico a large panel (*n* = 872) of sequenced genomes of *L. monocytogenes* to further assess the prevalence and diversity not only of type II RM systems but also of alternative RM systems such as those of type I and IV. The strains were selected based on the availability of adequate-quality whole genome sequence data (N50 > 35,000) from strains in our lab collection and with the intent of maximizing diversity based on source, isolation date, ST, CC, lineage and serotype.

**Figure 2:**
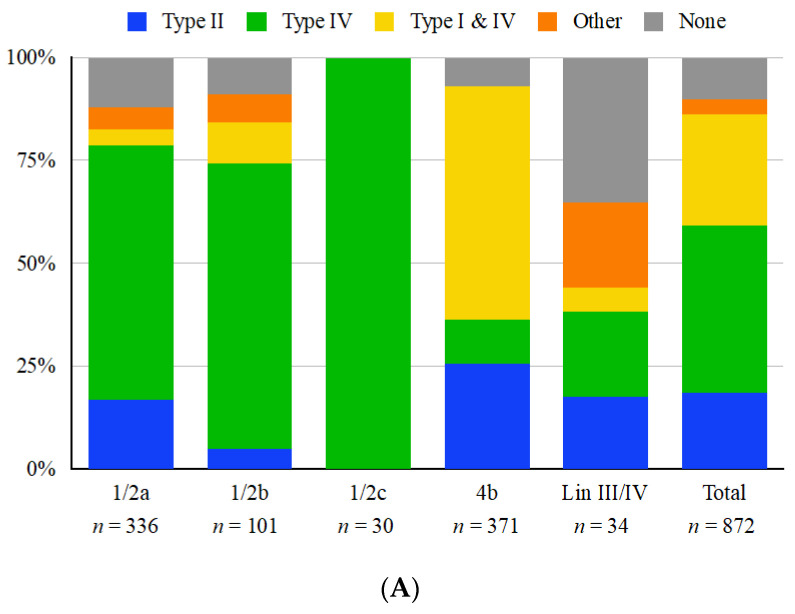

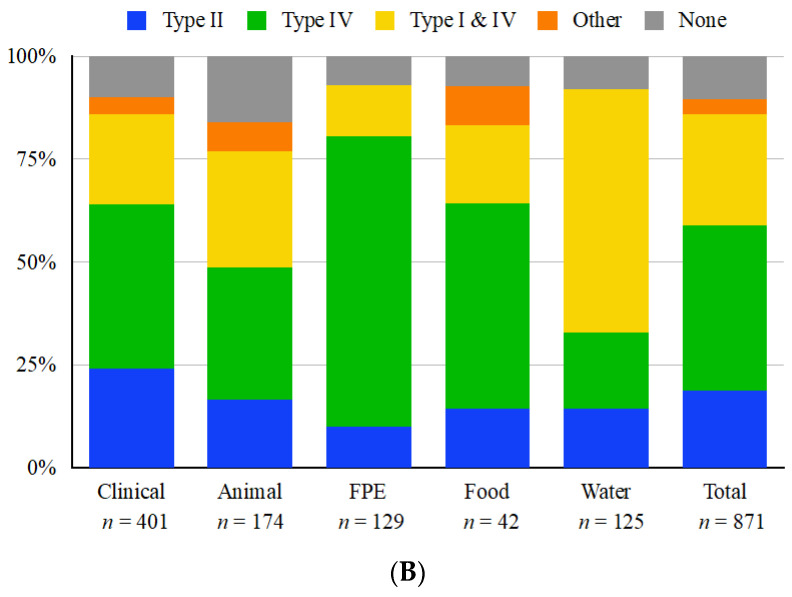
Distribution of restriction modification systems harbored in the immigration control region (ICR) located between *lmo0301* and *lmo0305* homologs among whole-genome sequenced *L. monocytogenes* strains. Distributions are shown by (**A**) serotype or lineage and (**B**) source. “Other” ICR content includes miscellaneous genomic islands and genes (e.g., DNA helicase genes) while “none” indicates no novel gene content, typically a lipoprotein and small hypothetical proteins. “Lin III/IV” indicates strains of Lineage III or IV (Table S1) for which accurate serotype designations are lacking. “Clinical” indicates strains of human clinical origin, while “Animal” includes strains from animal listeriosis as well as from wildlife, i.e., black bears (Table S1). A single strain (OLM81) has no known source and was excluded from panel B.

Of the 872 *L. monocytogenes* genomes in the panel, 760 (87.2%) harbored at least one type I, II or IV RM system in the ICR, while type III RM systems were not identified (Figure 2A). Analysis of the ICR content revealed that the different RM systems were not distributed homogeneously. Specifically, type II systems were mostly encountered in strains of serotypes 1/2a (lineage II) and 4b (mostly of lineage I), while the simultaneous presence of both type I and IV systems was uncommon in serogroup 1/2 but figured prominently among serotype 4b strains (Figure 2A). The reverse pattern was noted regarding type IV systems unaccompanied by type I, which were extensively encountered in serogroup 1/2 but were infrequent in 4b (Figure 2A). RM systems in the ICR were more commonly (>87.7%) encountered in the two major *L. monocytogenes* lineages (I and II) but were less common (55.9%) in strains of lineage III and IV (Figure 2A). A type IV *mcrB*-like restriction endonuclease was the most common system harbored in the ICR, being found in 61.9% of strains including 92 STs of diverse serotypes (1/2a, 1/2b, 1/2c, 4b) and lineages (I, II, III, IV) (Table 1 and Table S1). While type I RM systems in the ICR were found exclusively in conjunction with type IV Mrr or *mcrB*-like type IV systems, they were never encountered alone; furthermore, type II RM systems never co-localized with type I or IV systems (Figure 2A). All serotype 1/2c genomes harbored *mcrB*-like type IV systems, indicating that this may be a serotype-specific trait (Figure 2A). However, this may be due to the rarity and lack of diversity of serotype 1/2c strains in our panel (*n* = 30), all of which belonged to CC9 or CC789 (Table S1).

We were unable to identify strong links between isolation source and presence of RM systems (R-Squared < 0.065) (Figure 2B). However, approx. 59% strains from water were found to harbor type I and IV RM systems in their ICR, noticeably more than strains from other sources (Figure 2B; Table S1). This may reflect the fact that a majority of the water-derived strains were serotype 4b, namely the water-associated STs 217, 382, 639 and 663 [46,47]. As indicated above, serotype 4b strains frequently harbored type I and IV systems in the ICR. These higher levels of type I and IV RM systems in serotype 4b water isolates (81.2%) as compared to other source types (≤58.2%) may indicate a selective pressure unique to aquatic environments. Natural environments such as surface waters remain undersurveilled, but recent studies have shown a large amount of diversity [47,48,49].

### 3.2. ICR Presence and Content Are Completely Conserved within Each Sequence Type

**Figure 3:**
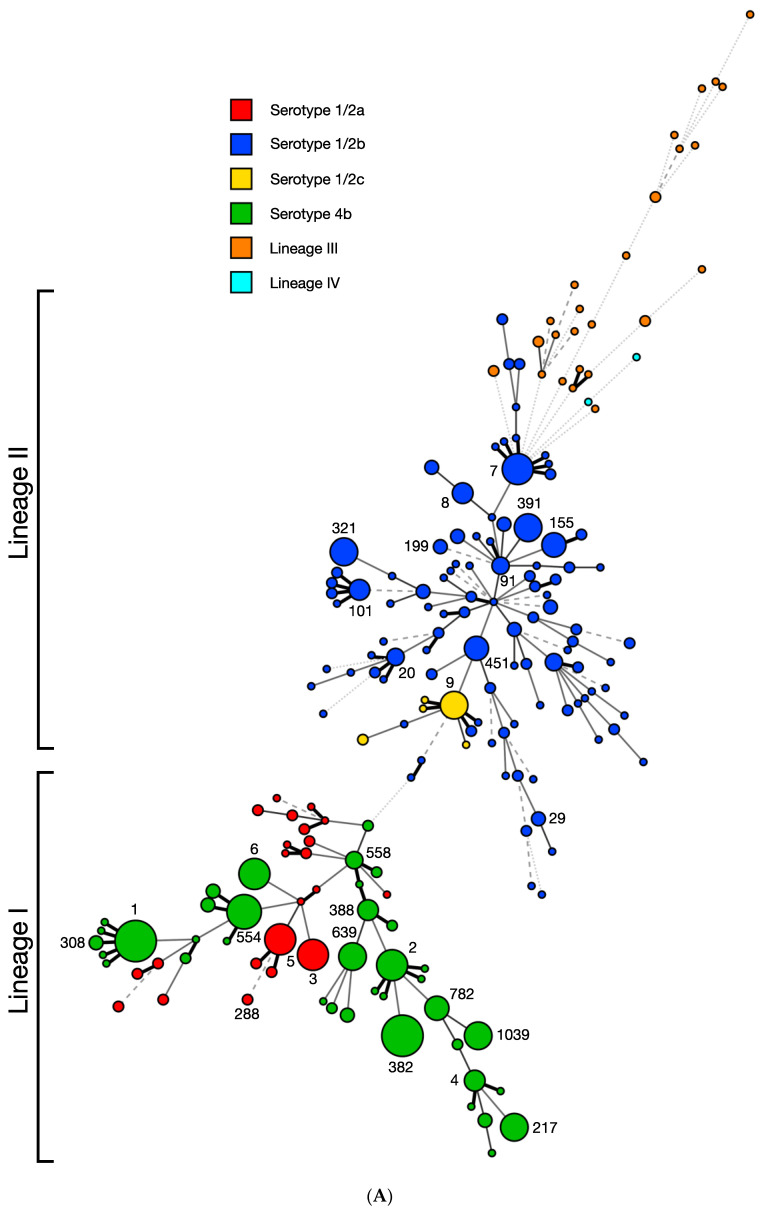

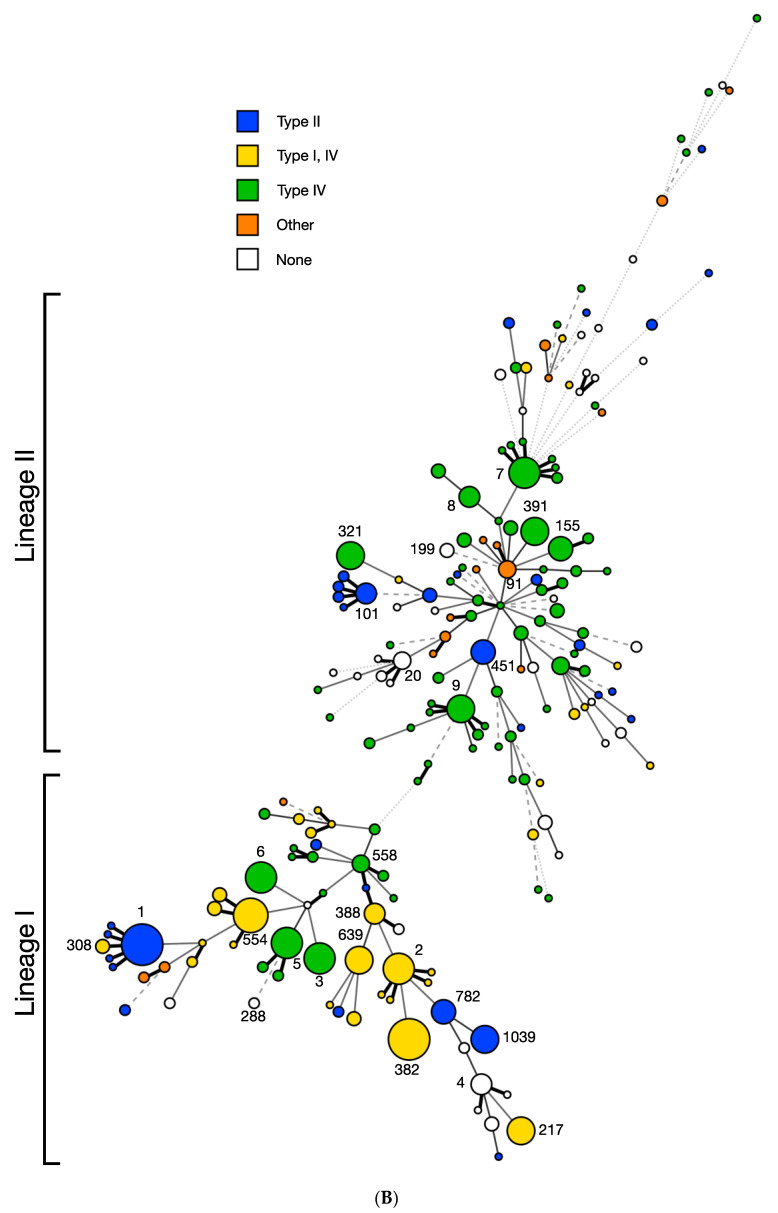
Distribution of restriction modification systems in the ICR in strains of diverse STs. Minimum spanning tree (MST) was constructed with the seven-locus MLST scheme using BioNumerics 8.1 as described in Materials and Methods. Each circle corresponds to a single sequence type (ST) and the circle size correlates to the number of strains in the respective ST; numbers next to circles denote relevant STs (e.g., 1, 2, 388, 1039). The smallest circles include one genome while the largest circle includes 50 genomes. STs are separated by branches based on seven-locus MLST similarity: one-allele difference (thick black line), two-allele differences (thin gray solid line), three-allele differences (dashed line), four-allele differences (dotted line), more than four-allele differences (thin dotted line). STs are color-coded based upon (**A**) serotype or lineage and (**B**) ICR content, specifically the harbored type of RM system(s). “Other” ICR content includes miscellaneous genomic islands and genes (e.g., DNA helicase genes), while “none” indicates no novel gene content, typically a lipoprotein and small hypothetical proteins.

The 872 *L. monocytogenes* included 192 sequence types (STs) based on the seven-locus multilocus sequence typing (MLST) scheme, distributed among different serotypes, with most STs belonging to lineage I or II (Figure 3A). Lineage III or IV consisted of a number of highly diverse STs (Figure 3A), in agreement with the previously documented pronounced genetic diversity of these uncommon lineages [17,18,19,50]. 

Remarkably, analysis of the 91 STs represented by more than two strains revealed that, without exception, presence/absence and content of the ICR were completely conserved within each ST (R-Squared = 1.0000, *p* < 0.0001) (Figure 3B). A striking example is the ubiquitous, ancient serotype 4b clone ST1 for which, as noted above, all of the investigated genomes harbored a Sau3AI-like type II RM system in the ICR, confirming previous findings from smaller strain panels [30]. The complete intra-ST conservation in ICR content suggests that these RM systems were likely acquired by the earliest member of the ST-defined clone and may have promoted clone emergence and stability in ways that remain to be elucidated. The GC content of these RM systems is significantly lower (27–35%) than the *L. monocytogenes* chromosomal average (38%) [51] (*p* < 0.0001), suggesting that they have been acquired by *L. monocytogenes* via horizontal gene transfer from other low-GC-content microorganisms such as *Clostridium*, *Bacillus* or *Staphylococcus*. (Table 1). 

As indicated above, the ICR content appeared to be completely conserved among all investigated strains of the same ST and was generally conserved even at the level of the same clonal complex. However, notable exceptions exist, including one of the six STs (ST308) in CC1 and two STs (782 and 1039) in CC2 (Table S1). Highly homologous type II RM systems such as Sau3AI-like and LmoJ3 were identified in unrelated STs and different lineages (Table 1), supporting the notion that these systems may be mobile but become stable once established in a clone. In *L. monocytogenes*, a strict clonal association of a RM system has only been shown for the type II RM system LmoH7, localized outside the ICR and conserved across all CC6 strains [31]. Additionally, certain other hotspots (e.g., guaA—*lmo1096*) have a propensity to harbor diverse genetic elements, including RM systems and the *Listeria* pathogenicity island-3 (LIPI-3) [33,38]. However, the *guaA* hotspot does not show complete clonal conservation, as certain ST5 strains harbor a Tn*916*-like transposon in this hotspot while others do not [52]. In this regard, the ICR chromosomal hotspot is novel, as it harbors diverse gene cassettes with complete intra-ST conservation.

### 3.3. Serotype 4b Strains Commonly Harbor Paired Type I and IV Restriction Modification Systems

As indicated above, Type I and IV systems were commonly (56.6%) found paired together in the ICR in strains of serotype 4b, where they accounted for the majority of RM systems, while this pairing was uncommon (5.0%) in other serotypes (Figure 2; Table S1). These RM systems were conserved not only within ST but also within CC and even serotype. For instance, nucleotide alignments between four strains of different STs within CC2, i.e., PNUSAL002849 (ST2), OLM11 (ST48), 4b1 (ST145), and OLM144 (ST2998), revealed 100% nucleotide similarity for these RM systems.

Investigation of the type I and IV RM systems from different CCs of serotype 4b revealed both conserved and divergent genes in these cassettes. Analysis of the paired type I and IV RM systems in the ICR of diverse STs of serotype 4b such as ST2 (CC2), 388 (CC388), 554 (CC554) and 663 (CC663) revealed high homology (≥98% nucleotide identity, 100% coverage) in five of the six genes in these two RM systems, with noticeable divergence being only noted in the type I RM system specificity subunit (Table 1). The latter only had approx. 58% nucleotide identity across these four serotype 4b STs, suggesting that, despite their overall similarities, these type I RM systems likely targeted different DNA sequences. Based on analysis using HHpred and BLASTp, all type I RM systems in our strain panel were predicted to be similar to EcoKI (Table 1). The type I RM system restriction (*hdsR*) and methylase (*hdsM*) subunits had ≥96.8% nucleotide conservation among the different *L. monocytogenes* genomes, with only the specificity subunit (*hdsS*) exhibiting high sequence diversity (28.5%) (Table 1). Type I RM system specificity changes in the presence of conserved endonuclease or methyltransferase genes have been previously described, with such changes shown to be inducible by transfection of an alternative type I RM system with a different specificity subunit [53,54]. Conserved genes in this cassette could also be found in other serotypes, albeit with higher divergence (Table S1). Strain 3623R (serotype 1/2a, ST14), a human clinical isolate from Sweden, was noteworthy in harboring a highly divergent (<50% nucleotide identity) form of the type I RM EcoKI-like system, flanking the ICR between *lmo0293* and lmo0294, as compared to the system found inside the ICR in other strains (Table S1). 

All type IV systems were predicted to be similar to AspBHI or McrB based on HHpred and BLASTp (Table 1). However, there was sequence diversity between similarly annotated systems in different strains; both the type IV AspBHI-like and McrB-like systems were each conserved at approx. 95% at the nucleotide level (Table 1). Similar levels of conservation were found between type IV systems which were found alone inside the ICR or were paired with a type I RM system (Table 1), suggesting that the type I RM systems were more recently acquired or were lost in the ICR.

### 3.4. Type II Restriction Modification Systems Can Be Found Both inside and Flanking the ICR

As indicated earlier, type II RM systems were encountered in 163/872 (18.7%) of the strains, primarily serotypes 1/2a and 4b in lineage II and I, respectively, as well as in lineage III/IV (Figure 2 and Figure 3). Furthermore, seven type II RM systems were identified: the widely encountered Sau3AI-like RM system targeting GATC sites and dependent on cytosine methylation, a MboI-like system (also targeting GATC sites but dependent on adenine methylation), NgoPII-like and HaeIII-like systems (both targeting GGCC), a SalI-like system (targeting GTCGAC), LmoJ2 (targeting GCWGC), and LmoJ3 (targeting GCNGC) (Table 1). Taken together, 41.3% of the strains harbored at least one type II RM system in or adjacent to the ICR (Table S1). A previous study found type II RM systems to be the most common RM system type in *L. monocytogenes* genomes, being detected in 80.5% of the genomes [34]. The lower prevalence of type II RM systems in or panel suggests that many other type II RM systems are harbored in chromosomal locations other than the ICR or its flanking sequences.

As indicated earlier, a Sau3AI-like type II RM system was previously reported to be conserved in the ICR of all tested strains of ST1 and other strains [30,55] (Figure 1). Our analysis confirmed that this RM system was harbored in the ICR of all ST1 strains and diverse STs of serotypes 1/2a, 1/2b, 4b and lineage III (98% + nucleotide identity, 100% coverage) (Table 1). Distinct and highly divergent (<50% nucleotide identity) cassettes also annotated as Sau3AI-like systems were found in the ICR-flanking region between *lmo0305* and *lmo0314* homologs in certain strains of serotype 1/2a, 4b and lineages III and IV (Table 1 and Table S1). Furthermore, the GATC-targeting MboI-like systems harbored by strains of serotypes 1/2b (ST3) and 4b (ST554 and CC558) were found on a gene cassette also harboring a Mu-like prophage gene gp29 in the ICR-flanking region, between *lmo0295* and *lmo0296*. Genomic DNA digestions of several of these strains indicated that they were resistant to MboI but were susceptible to Sau3AI digestion, suggesting that the MboI-like system in these STs is functional (R. M. Siletzky and S. Kathariou, unpublished findings) [55]. As noted earlier, Sau3AI and MboI-like RM systems both target GATC sites by methylating adenines and cytosines, respectively [45,56]. In total, we found that 200/872 (22.9%) of *L. monocytogenes* strains in our panel harbored a GATC-targeting system (Sau3AI or Mbo-like) in or adjacent to the ICR. This high prevalence of GATC-targeting systems may explain why large (131–136 kb) lytic *Myoviridae* bacteriophages such as A511 and P100 harbor so few (*n* = 0–2) GATC sites, thus remaining able to infect strains harboring the corresponding RM systems [30]. This paucity of GATC sites in *Myoviridae* phages may drive the replacement of Sau3AI-like RM systems with other cassettes better equipped to provide resistance to these phages.

The type II RM system LmoJ2 was found to be harbored by certain strains of serotype 1/2a and lineage III with high nucleotide conservation (99.6% + nucleotide identity, 100% coverage) (Table 1). Similarly, the type II RM system LmoJ3 was found in certain strains of serotype 1/2a and 4b with high nucleotide conservation (99.0% + nucleotide identity, 100% coverage) (Table 1). LmoJ2 (GCWGC) and LmoJ3 (GCNGC) recognition sites were found to be significantly less frequent in lytic *Siphoviridae* and *Myoviridae* bacteriophages, including A511 and P100 [32]. Additionally, these recognition sequences are not evenly distributed throughout the phage genome and are instead mostly found in certain genes, namely the tape measure protein and tail lysin [32].

The type II SalI-like RM system targeting GTCGAC was found exclusively in certain strains of lineage I (Table 1). Specifically, it was conserved in specific STs of CC2 (2, 48, 145) (serotype 4b) as well as in ST288 (CC288, serotype 1/2b) (Table S1). This SalI-like system was found downstream of the ICR between *lmo0318* and *lmo0319* (Table 1). While much less common, the type II RM NgoPII and HaeIII-like systems, both with the GGCC recognition motif, were found adjacent to the ICR in certain strains of lineage III, serotype 1/2a and serotype 1/2b (Table 1 and Table S1). NgoPII-like cassettes were found almost exclusively in historical strains isolated from 1933 to 1953 (Table S1), suggesting that this RM system may have been replaced with other cassettes in more modern strains or that the descendants of these strains are no longer commonly isolated.

Type III RM systems are characterized by methyltransferase and restriction endonuclease proteins, which form a single complex [57]. This protein complex targets two inversely oriented non-palindromic sequences and cleaves the DNA approx. 25 nucleotides upstream of the recognition sites [57]. Such systems were not encountered in the ICR and were detected only once adjacent to the ICR. Specifically, the serotype 4b strain CFSAN048783 of ST2 and isolated from apples in 2015 harbored a StyLTI-like type III RM system between *lmo0293* and *lmo0294* homologs (Table 1 and Table S1). Type III systems were previously reported in approx. 8% of 318 analyzed genomes [34]. Thus, the available data suggest that type III RM systems are uncommon in *L. monocytogenes* and, when present, are likely to be localized outside the ICR. Analysis of one of the strains (N1-011A) previously reported to harbor a type III RM system [34] indicated that the type III RM system was indeed outside the ICR. 

### 3.5. DNA Helicase and Other Diverse Genes Are Occasionally Found in the Immigration Control Region

While RM systems were by far the most commonly identified determinants in the ICR, certain strains harbored other gene cassettes in this region. Our analysis also revealed a number of STs that uniformly lacked RM systems in the ICR (Figure 3), with many of these strains instead harboring a lipoprotein found to be conserved in many strains of lineages I and II. Putative DNA helicase genes were found in the ICR in *L. monocytogenes* strains of serotype 1/2a, 1/2b and lineage III (Figure 1; Table S1). Further analysis by RAST, HHpred and REBase failed to suggest any additional putative functions of these genes. DNA helicase genes in the ICR were commonly (20/30—66.7%) flanked by type II RM systems, such as NgoPII-like and Sau3AI-like systems (Table S1). Putative DNA helicase genes were actually found to be part of the type IV restriction system SauUSI in *Staphylococcus aureus* [29]. Bacteriophage resistance genes are not uniformly distributed throughout the chromosome and are typically clustered together into defense islands even when they are not functionally related, e.g., restriction modification systems and toxin-antitoxin systems [58,59]. Further analysis would be required to understand the potential roles of these putative DNA helicase genes in the ICR.

Major facilitator superfamily (MFS)-type transporters were identified in the ICR in two strains of lineage III (Table S1). These determinants can mediate the transport of diverse substrates in or out of the cell, and in *L. monocytogenes* and other Gram-positive bacteria they can function as multidrug efflux pumps, increasing tolerance to benzalkonium chloride and lincomycin [60,61,62]. While evidence of the potential roles of MFS-type transporters in bacteriophage resistance is currently lacking, their localization in the ICR provides compelling reasons for their further investigation.

Putative DNA-adenine methyltransferases without cognate endonuclease genes were also identified in the ICR-adjacent region in certain strains of *L. monocytogenes*, specifically in the serotype 1/2a strains of CC31 and ST935 (Table 1 and Table S1). Additional work is needed to understand the functions of these genes, but their proximity to a defense island hotspot suggests their possible involvement in bacteriophage resistance [58,59].

Several of the genomes, primarily in lineage III/IV and less commonly in serotypes 1/2a, 1/2b and 4b, appeared to lack any additional novel content in their ICR, with these strains generally harboring a single lipoprotein and small hypothetical proteins (Figure 2A and Figure 3B). This lack of recognizable ICR content was a clonal trait, being noted among all investigated strains of the same ST and CC. Most notably, the strains of the hypervirulent CC4 uniformly lacked RM systems in the ICR, instead harboring a lipoprotein and a small 288bp hypothetical protein with no known function (Table S1). Additionally, strains of CC4 lacked RM systems in the region flanking the ICR (Table S1). Other notable STs which harbored no RM systems in the ICR are the serotypes 1/2a STs 20, 29 and 37; however, these strains harbor a Sau3AI-like system in the region flanking the ICR between *lmo0305* and *lmo0314* (Table S1). The absence of ORFs in this region, especially in CC4, may suggest a selection for loss of RM systems in this region.

## 4. Conclusions

We found that RM systems are commonly harbored in and adjacent to the ICR, highlighting the importance of the region for further study to better understand the mechanisms employed by *L. monocytogenes* to protect itself against foreign DNA (e.g., bacteriophage, plasmids and transposons) and to preserve its genomic integrity. The low GC content of the RM systems in the ICR suggests that they were mobilized into *L. monocytogenes* via horizontal gene transfer from other organisms; however, the intraclonal conservation of ICR gene content indicates that, once established, these systems are highly stable. This conservation highlights the importance of the ICR in promoting clone emergence and stability at the ST level.

The type I EcoKI-like and type IV *mcrB*-like systems were the most common RM systems in the ICR (Table 1) and were commonly found paired together, especially in strains of serotype 4b (Figure 2). While high diversity was found in the EcoKI-like *hdsS* subunit, overall the greatest diversity was found in type II RM systems (Table 1). GATC and GCNGC-targeting systems were the most common type II RM systems in our panel. Previous studies have found that the frequency of GATC and GCNGC recognition sites is significantly lower in the genomes of lytic phages than in temperate phage genomes or the *Listeria* chromosome, suggesting that such sites have been selectively eliminated to circumvent these common RM systems [30,32]. Additional work is warranted to better understand the roles of these systems in the ecology and evolution of *L. monocytogenes*, especially in terms of bacteriophage resistance and acquisition of foreign DNA.

## Figures and Tables

**Table 1:** Distribution and conservation of restriction modification systems harbored in and adjacent to the ICR in the analyzed *Listeria* genomes. Canonical recognition sites are in parentheses.

Restriction Modification System ^1^	Location	Incidence	Number of Strains	Conservation (nt Identity %) ^2^	GC Content (%)
**Type I: EcoKI-like** (AACN_6_GTGC)	*lmo0301*-*lmo0305*	1/2a, 1/2b, 4b, lineage III	235	*hdsR*: ≥98.0 *hdsM*: ≥96.8 *hdsS*: ≥28.5	35
**Type I: EcoKI-like** (AACN_6_GTGC)	*lmo0293-lmo0294*	1/2a	33	*hdsR*: 100 *hdsM*: 100 *hdsS*: 100	33
**Type II: HaeIII-like** (GGCC)	*lmo0305*-*lmo0314*	1/2b	9	100	27
**Type II: LmoJ2-like** (GCWGC)	*lmo0301*-*lmo0305*	1/2a, lineage III	15	≥99.6	32
**Type II: LmoJ3-like** (GCNGC)	*lmo0301*-*lmo0305*	1/2a, 4b	59	≥99.0	30
**Type II: MboI-like** (GATC)	*lmo0295-lmo0296*	1/2b, 4b	57	100	30
**Type II: NgoPII-like** (GGCC)	*lmo0305*-*lmo0314*	1/2a, lineage III	8	≥97.6	31
**Type II: SalI-like** (GTCGAC)	*lmo0318-lmo0319*	1/2b, 4b	35	99.9	31
**Type II: Sau3AI-like** (GATC)	*lmo0301*-*lmo0305*	1/2a, 4b, lineage III	89	≥98.0	31
**Type II: Sau3AI-like** (GATC)	*lmo0305*-*lmo0314*	1/2a, 4b, lineage III, lineage IV	53	≥48.8	31
**Type III: StyLTI-like** (CAGAG)	*lmo0293-lmo0294*	4b	1	NA	31
**Type IV Mrr: AspBHI-like** (YSCNS)	*lmo0301*-*lmo0305*	1/2a, 1/2b, 4b	100	≥97.0	31
**Type IV *mcrB*-like** (R^m^C)	*lmo0301*-*lmo0305*	1/2a, 1/2b, 1/2c, 4b, lineage III	540	≥95.0	30

^1^ N (any base), Y (C or T), S (G or C), R (A or G). ^2^ Conservation refers to comparisons among strains of different sequence types.

## Data Availability

Accession numbers for whole genome sequenced strains used in this study are listed in Table S1 and are publicly accessible at the National Library for Biotechnology Information (NCBI).

## Supplementary Materials

The following supporting information can be downloaded at: https://www.mdpi.com/article/10.3390/microorganisms11030699/s1. Table S1. *Listeria monocytogenes* strains used in this study and their relevant characteristics including immigration control region (ICR) content. NA indicates that no restriction modification systems were found in the ICR flanking regions.
